# Dendrimers and Dendritic Materials: From Laboratory to Medical Practice in Infectious Diseases

**DOI:** 10.3390/pharmaceutics12090874

**Published:** 2020-09-14

**Authors:** Miguel Ángel Ortega, Alberto Guzmán Merino, Oscar Fraile-Martínez, Judith Recio-Ruiz, Leonel Pekarek, Luis G. Guijarro, Natalio García-Honduvilla, Melchor Álvarez-Mon, Julia Buján, Sandra García-Gallego

**Affiliations:** 1 Department of Medicine and Medical Specialities, Faculty of Medicine and Health Sciences, University of Alcalá, 28801 Alcalá de Henares, Spain; miguel.angel.ortega92@gmail.com (M.Á.O.); aguzmanmerino@hotmail.com (A.G.M.); oscarfra.7@hotmail.com (O.F.-M.); leonel.pekarek@gmail.com (L.P.); natalio.garcia@uah.es (N.G.-H.); melchor.alvarezdemon@uah.es (M.Á.-M.); mjulia.bujan@uah.es (J.B.); 2 Institute Ramón y Cajal for Health Research (IRYCIS), 28034 Madrid, Spain; 3 Tumour Registry, Pathological Anatomy Service, University Hospital Príncipe de Asturias, 28805 Alcalá de Henares, Spain; 4 University Center for the Defense of Madrid (CUD-ACD), 28047 Madrid, Spain; 5 Department of Organic and Inorganic Chemistry, Faculty of Sciences, and Research Institute in Chemistry “Andrés M. del Río” (IQAR), University of Alcalá, 28801 Alcalá de Henares, Spain; judithrecio1997@gmail.com; 6 Department of Systems Biology, Faculty of Medicine and Health Sciences, University of Alcalá, 28801 Alcalá de Henares, Spain; luis.gonzalez@uah.es; 7 Networking Research Centre on Hepatic and Digestive Diseases (CIBER-EHD), 28029 Madrid, Spain; 8 Immune System Diseases-Rheumatology, Oncology and Medicine Service, University Hospital Príncipe de Asturias, 28805 Alcalá de Henares, Madrid, Spain

**Keywords:** dendrimer, nanoparticle, infection, bacteria, virus, fungi, parasite, amoeba, prion

## Abstract

Infectious diseases are one of the main global public health risks, predominantly caused by viruses, bacteria, fungi, and parasites. The control of infections is founded on three main pillars: prevention, treatment, and diagnosis. However, the appearance of microbial resistance has challenged traditional strategies and demands new approaches. Dendrimers are a type of polymeric nanoparticles whose nanometric size, multivalency, biocompatibility, and structural perfection offer boundless possibilities in multiple biomedical applications. This review provides the reader a general overview about the uses of dendrimers and dendritic materials in the treatment, prevention, and diagnosis of highly prevalent infectious diseases, and their advantages compared to traditional approaches. Examples of dendrimers as antimicrobial agents per se, as nanocarriers of antimicrobial drugs, as well as their uses in gene transfection, in vaccines or as contrast agents in imaging assays are presented. Despite the need to address some challenges in order to be used in the clinic, dendritic materials appear as an innovative tool with a brilliant future ahead in the clinical management of infectious diseases and many other health issues.

## 1. Introduction

Infectious diseases are produced by pathogenic microorganisms, mainly bacteria, viruses, parasites, and fungi. Since the dawn of civilization, these diseases have persisted as sources of human morbidity and mortality, representing the second cause of death worldwide and the main reason of disability-associated reduction in quality of life. These diseases have important economic repercussions and affect both developed and developing countries [1]. Likewise, emerging pathogens are a major public health problem which could become a pandemic as is currently happening with COVID-19, and it is important to prevent their appearance [2]. 

Numerous social factors are directly related to the appearance and development of infectious diseases such as the lack of education, housing, and employment; a low socioeconomic level; reduced hygiene; water scarcity or malnutrition [3]. Furthermore, recent studies showed the impact of climate change in the dissemination and transmission of infectious diseases, as well as in the appearance of vector-borne diseases and re-emergences, i.e., extinct or very marginal infections which are reappearing in a more virulent form or in a new epidemiological framework, increasing exponentially both in their incidence and in their geographical range [4,5]. Some other examples of well-known effects of climate change are the rising ocean levels or the increased air pollution. The first of these factors implies smaller fields of cultivation, which could lead to the undernourishment of certain populations, and the second is related to acute respiratory diseases, especially asthma. These effects on human health would make people more fragile and prone to developing infectious diseases by creating an environment more conducive to the transmission, appearance, and re-emergence of these diseases. In addition, globalization, mobility between countries, or behavioral patterns specific to each region of the world can be important in the transmission of established or new infectious diseases [6].

The development of preventive measures is one of the most important tools to manage infectious diseases. The role of governments in the establishment of public health laws, including national vaccination programs, is fundamental [7,8]. Similarly, rapid diagnosis of infectious diseases can play an important role in the control of these pathologies; however, this is currently a challenge, especially in countries with limited economic resources [9]. According to the World Health Organization (WHO), the development of effective diagnostic tests must comply the “ASSURED” criteria: Affordable, Sensitive, Specific, User-friendly, Robust and rapid, Equipment-free, and Deliverable to those who need them [10]. Some authors also propose the importance of introducing digital and portable technologies for more accurate and detailed information on the disease [9]. The development of tests and diagnostic techniques may also have other benefits beyond the individual patient’s own diagnosis, such as the screening of high-risk groups, the study and surveillance of epidemics and outbreaks, or the monitoring of the treatment received by the patient. For these reasons, further development of more accurate and practical diagnostic systems is imperative in the management of infectious diseases worldwide.

Antimicrobial drugs are the key tool in the treatment of infected patients. However, their indiscriminate and inappropriate use by the general population, and the inadequate medical or pharmaceutical practice have promoted the appearance of resistant microorganisms, which are associated to high morbidity and mortality [11]. Considering the scarcity of effective therapies and preventive measures, the opening of new therapeutic windows is crucial to curb this public health problem [12], as well as adequate public health policies and resources [13]. Antimicrobial resistance has an outstanding impact on the development of nosocomial infections. These are defined as local or systemic processes resulting from an adverse reaction to the presence of an infectious agent or its toxins, which was neither present nor incubating upon admission to the health care facility. To combat these threats, a number of institutions have emerged, such as the European Centre for Disease Prevention and Control (ECDC, https://www.ecdc.europa.eu/en), which aims to support European countries in the fight against infectious diseases. Thus, 17 epidemiological surveillance networks have been developed which coordinate the monitoring of antimicrobial resistance between Member States. However, it seems necessary to expand its coverage to a wider spectrum of antimicrobial agents, as well as to increase the number of laboratories participating in these networks.

Overall, it is essential to develop more effective preventive, diagnostic, and therapeutic strategies. In this context, the use of nanoparticles is a promising tool to control and manage infectious diseases. Nanotechnology is an interdisciplinary area that encompasses aspects of various scientific fields such as biology, chemistry, and physics, and which has experienced an extraordinary growth since the 1990s. It is based on the design of nanometric materials (nanoparticles, nanorods, or nanowires, among others) that provide very different properties to those of the macroscopic material, such as their ability to be artificially customized and the high surface-to-area ratio [14]. Thanks to these unique properties, they are very useful in the field of biomedicine, leading to a growing field of interest known as nanomedicine, which employs these tools as a support or mean of interaction with cells and tissues both *in vivo* and *ex vivo*, enabling a wide variety of applications in multiple pathologies [15]. In the case of infectious diseases, nanomaterials have been employed as antimicrobial agents, as imaging tools and rapid diagnostic techniques [16], in the development of vaccines or even in the modulation of the immune response [17]. Furthermore, nanoparticles based on biocompatible and biodegradable materials, such as lipids or natural polymers, can reduce the toxicity associated with drug administration in infectious diseases [18].

Nevertheless, nanomaterials must overcome several challenges related to their high costs, innovation, and quality, before achieving a real impact on human health. There is a wide variety of different nanostructures that can be used in the field of biomedicine. Among the most promising systems are iron oxide magnetic nanoparticles [19], polymeric nanogels [20], and dendrimers [21]. 

This review provides the non-specialist reader an introduction into the field of dendrimers and dendritic materials as well as a complete overview of their impact and potential on the treatment, prevention and diagnosis of prevalent infectious diseases, caused by virus, bacteria, fungi, parasites, amoebas, and prions.

## 2. Dendrimers: Synthesis, Uses, and Challenges to Face in Biomedicine

### 2.1. Dendrimers

Dendrimers are a class of polymeric molecules discovered in the 1970–1980s by research teams led by scientists such as Tomalia, Vögtle, Denkewalter, and Newkome [22]. Their name derives from the Greek word “dendron”, meaning tree, which refers to their branched structure. Dendrimers and dendrons belong to the family of dendritic polymers and stand out due to their controlled synthesis and monodispersity, i.e., they always present exactly the same structure [23]. Their size ranges between 1–10 nm, depending on their generation, and they have a three-dimensional hyperbranched architecture which confers substantial differences compared to linear polymers, such as:

- The ability to customize and control the size and shape of the dendrimer through the synthetic process; this enables researchers to fit their design to their purpose, for example by attaching drugs, antibodies or imaging probes in specific positions of the nanoparticle.
- The possibility to cross cell membranes due to their controllable size and, in some occasions, assisted by the lipophilicity of the skeleton.
- The possibility of encapsulating drugs and targeting them to the desired tissue, reducing the toxicity and providing greater control by simplifying its administration [24].
- A unique behavior of the intrinsic viscosity and glass transition temperatures, explained by the absence of entanglement at higher molecular weights.

The above-mentioned properties make dendrimers highly attractive in the field of biology and medicine, including infectious diseases.

**Figure 1:**
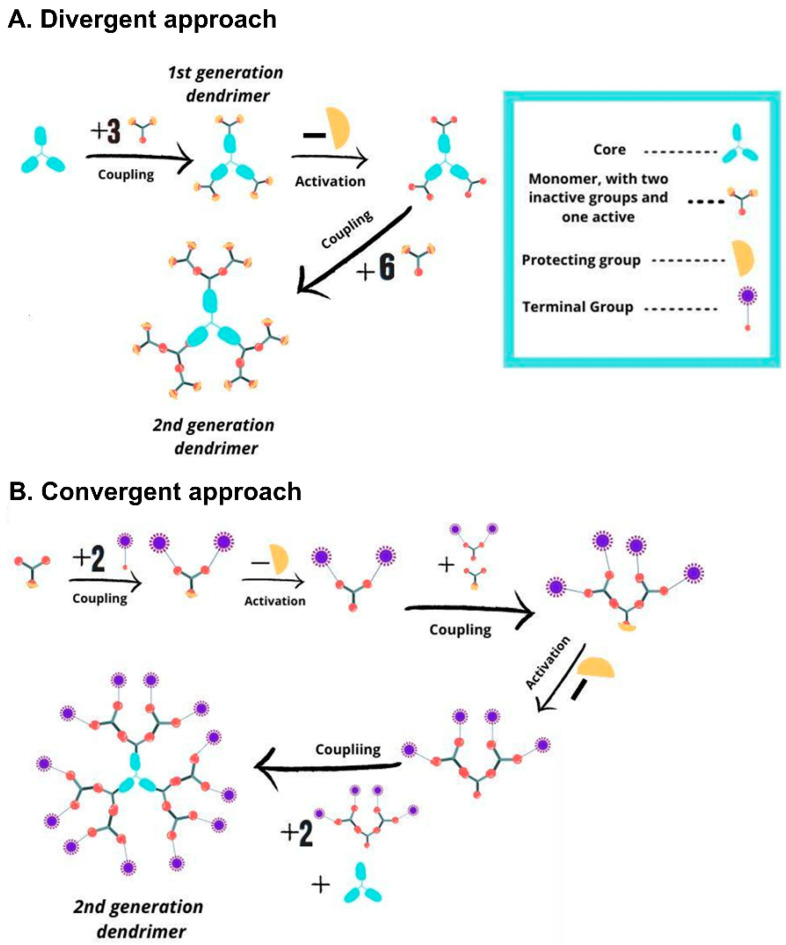
Scheme depicting the traditional synthetic approaches to dendrimers. (**A**) Divergent growth approach, inside-out strategy from the core. (**B**) Convergent growth approach, outside-in strategy from the terminal groups.

Dendrimers consists of three main domains: (1) The core, located in the center of the dendrimer, which can comprise one or multiple atoms; (2) the branching units, which are covalently linked to the core, whose repetition leads to a series of dialectically-concentric layers. The number of these layers is known as the dendritic “generation”; and (3) the terminal groups, mainly located on the surface of the dendritic scaffold, and highly responsible of the dendrimer properties [25]. Two “traditional” synthetic routes are prevalent (Figure 1): the divergent strategy and the convergent strategy [23]. In the divergent growth approach, first developed by Tomalia et al. [26], the dendrimer synthesis proceeds inside-out from the core. The core reacts with AB_n_ monomeric units through the A functional group, while the B groups are dormant/protected and will react in a subsequent step after deprotection/activation. Iterative growth and activation steps lead to the desired generation, while the end-groups are available for further postfunctionalization. The divergent growth is the most viable approach, as it employs an excess of inexpensive reagents, but could lead to structural defects at high generations due to incomplete substitutions. The convergent growth approach was initially developed by Hawker and Fréchet, to improve the weaknesses of the divergent approach. This outside-in strategy relies on the coupling of monomers to generate monodisperse dendrons, which are finally attached to a multifunctional core through their focal points. While the risk of structural defects is minimized, the synthesis of higher generation dendrons and dendrimers are challenging due to steric hindrance, leading to low yields. Importantly, new strategies, namely “accelerated growth” approaches, are continuously evolving to simplify the synthetic routes while keeping their perfection. These include the orthogonal chemoselective strategy and the one-pot approaches, among others. The number of steps is thus reduced, as the chemoselective moieties avoid the need for protection/deprotection steps. The reader is referred to excellent reviews on this topic [23,27].

#### 2.1.1. Main Dendritic Families in Biomedicine

**Figure 2:**
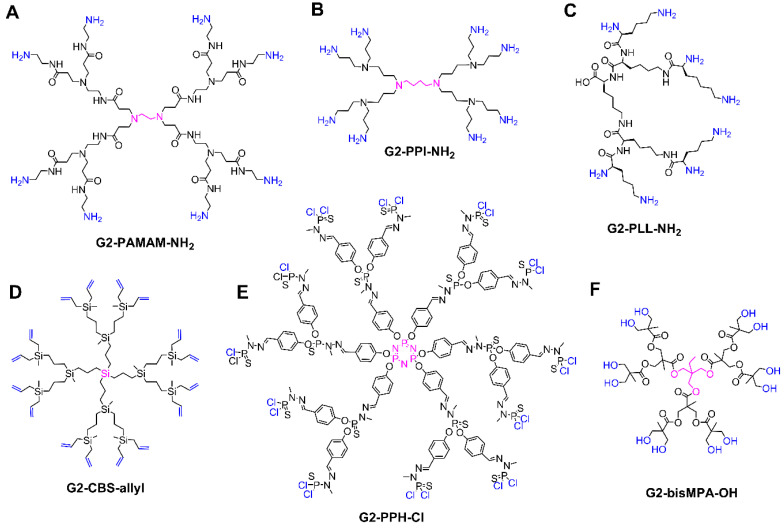
Overview of the most common dendritic families for biomedical applications, depicting the structure of second generation dendrimers. (**A**) Poly(amino amide) (PAMAM). (**B**) Poly(propylene imine) (PPI). (**C**) Poly(L-lysine) (PLL). (**D**) Carbosilane (CBS). (**E**) Poly(phosphorhydrazone) (PPH). (**F**) Bis-MPA polyester. The core is highlighted in pink, the terminal groups in blue.

A broad variety of dendritic scaffolds have been described in the literature, with a purpose-driven design. For details on their preparation, the reader is referred to excellent books and reviews on the literature [28,29,30]. In the biomedical field, the dendritic families which stand out are poly(amino amide) (PAMAM) [31], poly(propylene imine) (PPI), poly(L-lysine) (PLL) [32], carbosilane [33], poly(phosphorhydrazone) (PPH) [34], and polyester dendrimers [35] (Figure 2). The biocompatibility, flexibility, and commercial availability are behind their prevalence in this field. 

PAMAM dendrimers are probably the most studied dendritic architectures, reaching up to the tenth generation, with different cores and terminal groups (mainly NH_2_ or OH), Figure 2A. PAMAM dendrimers exhibit appealing properties for biomedical studies [31], such as a high water solubility, a peptide-mimicking backbone, and readily modifiable amine termini. 

PPI dendrimers, also known as POPAM or DAB, present multiple tertiary amines on the scaffold and primary amines as terminal groups, Figure 2B. They are comparatively smaller than PAMAM and present a more hydrophobic scaffold, but are also commercially available, prevalent in the biomedical field and similarly cytotoxic due to the peripheral amino groups [36].

PLL dendrimers, which comprise the amino acid lysine in their entire structure (Figure 2C), stand out due to their biocompatibility, biodegradability, and the ability to maintain its activity in environments with high and low salinity [32]. PLL differ from other dendrimers such as PAMAM and PPI in the asymmetry of their branching cell, which inevitably influences the encapsulation properties as they possess no interior void space [37]. However, they share the presence of multiple NH_2_ peripheral groups, which can cause certain cytotoxicity.

Carbosilane dendrimers comprise carbon–carbon and carbon–silicon bonds in their scaffolds, conferring flexible, non-polar, inert, and thermally stable properties, very interesting in biomedicine [33], Figure 2D. They are often decorated with polar groups in order to achieve water solubility. They are classified as “inorganic dendrimers” and exhibit relevant differences compared to traditional “organic dendrimers” such as PAMAM. Furthermore, they have a great variability by altering the core and the amount and length of the branches.

PPH dendrimers, which can be quantitatively prepared up to generation 12 [38], present phosphorus atoms throughout the entire dendritic scaffold and have been widely studied for biomedical applications [34], Figure 2E. Like carbosilane dendrimers, PPH are also “inorganic dendrimers” with a huge variability in cores, branches, and peripheral groups, and require the attachment of polar groups in the periphery to become water-soluble.

Polyester dendrimers attract widespread attention in the biomedical field due to their biocompatibility and biodegradability. In particular, dendrimers based on 2,2-bis(hydroxymethyl)propanoic acid (bis-MPA, Figure 2F) are commercially available. Since the first reports in the early 1990s, bis-MPA dendrimers have undergone an extraordinary increase in their structural complexity and control, which capitalized on a constant evolution of the synthetic strategies, from the traditional divergent and convergent routes to accelerated approaches based on chemoselective reactions [35,39].

#### 2.1.2. Main Uses of Dendritic Materials in Biomedicine

Since their first reports, dendrimers have been tested in a large number of *in vitro* and *in vivo* studies for multiple biomedical applications. The most explored use of dendrimers is their ability to carry drugs to the desired site of action, being an important resource in precision medicine [24]. Dendrimers protect the encapsulated or bound drug and allow the delivery to the desired site of action. As they can be customized, dendrimers improve the drug pharmacokinetics and solubility, control the drug release, enable more comfortable administration routes, and reach target sites with difficult accessibility such as the ocular system [40]. Another application that has raised great interest is the use of dendrimers in gene therapy. Several dendrimers have been explored as non-viral vectors for DNA and RNA, enabling gene transfection to specific cells. This has been especially useful in *in vitro* cancer studies, where RNA transfected to tumor cells can alter their mechanisms, making them more susceptible to treatment or hindering their uncontrolled division [41]. On the other hand, dendrimers can act as immunomodulators, by either reducing or enhancing the immune response [42]. The first approach can be very useful towards autoimmune diseases and allergies [43], while the second has been employed, for example, in cancer immunotherapy [44]. The attachment of multiple antigen copies to the dendritic scaffold produces an increase in the immune response related to the multivalency and the decrease in the conformational freedom of the antigen. In infectious diseases, dendrimers can support the development of vaccines by acting as antigens carrier, providing stability, safety, and a sustained release. In addition, they can serve as adjuvants or can promote the uptake of the antigen by the antigen-presenting cells, thus enhancing its recognition and improving the effectiveness of the vaccine [45].

Another outstanding application is the use of dendritic materials in diagnosis [46], such as iron oxide magnetic nanoparticles decorated with dendrimers, which can be monitored through magnetic resonance, or the oxygen sensors, very useful in pathologies such as diabetes [47]. Dendrimers also enable a combined therapeutic and diagnostic action in a single platform, the so-called “theranosis” [48]. The diagnostic capacity is provided by a specific molecule (e.g., a radionuclide) which, bound to the surface or encapsulated inside the nanoparticle, serves to detect its position *in vivo* by means of diagnostic imaging such as single photon emission computed tomography (SPECT). 

**Figure 3:**
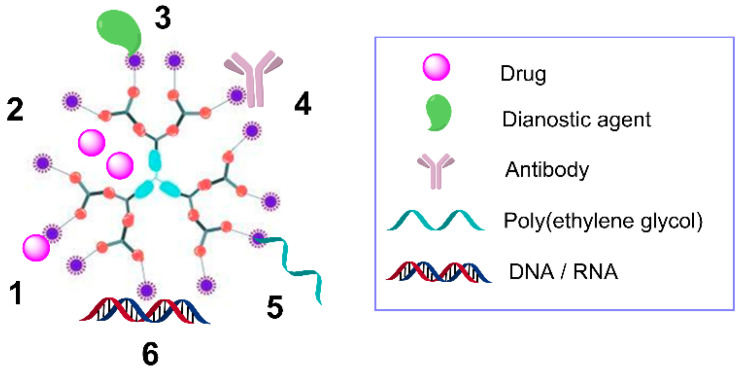
Scheme of a second-generation dendrimer and its interactions with multiple agents, including covalently-bound drugs (1), encapsulated drugs (2), covalently-bound diagnostic agents (3), antibodies (4), poly(ethylene glycol) (5), and electrostatic interaction with nucleic acids (6).

In order to fully benefit from the use of dendrimers as nanocarriers, it is essential to understand the mechanisms of interaction between the dendrimer and the different cargo (Figure 3) [49,50,51]:

- Encapsulation. The drug is physically trapped within the dendritic scaffold due to the spheroidal or ellipsoidal hollow cavities found between the different branches. These cavities are frequently hydrophobic, so they exhibit affinity towards drugs with poor water-solubility, and can also lead to H-bonding due to the presence of oxygen and nitrogen atoms. The main drawback of this approach is the tendency of the drug to rapidly leak in biological fluids, compared to a covalent conjugation approach [52].
- Electrostatic interactions. The multivalent structure of the dendrimer enables the formation of multiple bonds in the periphery, which depend on the nature of the end groups. A common example are electrostatic interactions between the drug and a dendrimer bearing cationic (e.g., ammonium groups) or anionic (e.g., carboxylate) moieties. PAMAM and PPI dendrimers frequently employ this mechanism, due to the multiple ionizable amino groups in the periphery as well as in the interior of their scaffolds. The pH, the ionic strength and the presence of proteins such as albumin have a remarkable impact on dendrimer–cargo electrostatic interactions [52]. This approach is widely employed in gene therapy to generate dendrimer–nucleic acid complexes, or “dendriplexes” [53].
- Covalent conjugation. Drugs and other molecules can be attached to dendrimers through covalent bonds. Sometimes labile or biodegradable bonds are employed, such as amide or ester bonds, to enable the release under chemical or enzymatic scission. Other strategy relies on the use of spacers, such as poly(ethylene glycol) (PEG), which also generates a hydrophilic surface with a hydrophobic interior, an amphiphilic unimolecular micelle to improve drug encapsulation. Furthermore, the attachment of PEG reduces the interaction with blood proteins and cells, prolongs the circulation in blood and increases the overall molecular weight, improving the permeability and retention of the drug [54]. Other types of ligands have also been covalently bound, such as antibodies or contrast agents. This type of interaction increases the stability of the drug towards degradation, alters the release kinetics, and improves the therapeutic efficiency.

These three strategies have also been exploited in the treatment of infectious diseases, as previously reported [55,56,57,58]. The present review, however, focuses on a broader overview to cover the prevention, treatment, and diagnosis of these diseases, as detailed in Section 3. 

#### 2.1.3. Commercial Potential of Dendrimers and Challenges to Face in Biomedicine

The interest in the dendrimer field has continuously increased over time. As recently reported by Tomalia (2020) [59], more than 60,000 articles/patents have been published on dendritic materials, with an approximate increase of 1500 publications and 3000 patents per year since 2013. Key commercial successes include the Stratus CS Acute Care Diagnostic System (Siemens Healthcare GmbH, Erlangen, Germany), for emergency diagnosis of cardiovascular infarctions; VivaGel^®^ products (Starpharma, Melbourne, Australia), for the prevention and treatment of sexually transmitted infections (STIs); Targeted DEP^®^ and Priostar^®^ (Starpharma), for the delivery of anticancer drugs and agrochemical products, respectively; or SpheriCal (Polymer Factory, Stockholm, Sweden), as mass spectrometry standards [59].

From the low rate of issued patents turning into commercial products, it becomes apparent that dendritic materials must face several challenges for the bench-to-bedside translation in the biomedical field. Mignani et al. (2017) summarized the requirements to become a clinical candidate [60]. To secure a successful development, the authors highlight the importance of complying with the Good Laboratory Practice (GLP) requirements to ensure the quality, reproducibility and reliability of *in vitro* and *in vivo* data. Furthermore, the Good Manufacturing Practice (GMP) is desirable, but emerges as one of the main challenges in dendrimer translation. Indeed, dendrimer defects have been related to the failure of relevant preclinical trials [61]. An accurate dendrimer synthesis and a thorough purification process are deemed necessary to ensure monodispersity and batch-to-batch reproducibility. This is a highly demanding challenge especially for high-generation dendrimers, multipurpose platforms, and large-scale production. Many different strategies are currently explored to overcome these challenges in dendrimer translation, including engineering through critical nanoscale design parameters (CNDPs) [37]; accelerated synthetic approaches [27]; the improvement of analytical techniques, such as mass spectrometry; the accurate and simplified design of multipurpose platforms [62]; or the dendronization of materials to expand their uses [63].

## 3. Dendrimers and Dendritic Materials in the Prevention, Treatment, and Diagnosis of Infectious Diseases

### 3.1. The Role of Dendritic Materials against Viral Infections

Viruses are simple acellular organisms, which have coevolved with living beings to replicate and reproduce inside their cells, after the binding to specific receptors [64]. Nearly 5000 species of viruses have been reported; many of them can cause human diseases [65]. Some viral infections, such as respiratory ones, represent an important economic burden and a serious public health concern [66]. Antiviral drugs are the most common clinical tool to address these pathologies. To date, 86 different drugs have been approved for the treatment of 17 viral infections [67]. However, some drugs have serious side effects, including nausea, insomnia, vomiting, allergic reactions, behavior disorders, cardiovascular complications, and dependency [68]. Opening new therapeutic windows, decreasing the side effects while maintaining their efficacy, is a key action. 

Dendrimers contribute to the fight against viral infections [69], acting as microbicides per se or as drug nanocarriers, with relevant properties such as low systemic absorption, biocompatibility, water solubility, or simple formulation [70]. The main uses of dendrimers in viral infections are herein addressed, the Human Immunodeficiency Virus (HIV) being one of their most important targets.

#### 3.1.1. Dendrimers and Dendritic Materials against HIV Infection

STIs are highly prevalent worldwide, despite the efficient preventive tools (e.g., preservatives) [71]. One of the most illustrative examples is the Human Immunodeficiency Virus (HIV). HIV is responsible for the deterioration of immune cells, especially the target CD4+ T cells [72], thus aiding the entry of opportunistic pathogens that cause the Acquired Immunodeficiency Syndrome (AIDS) [73]. HIV transmission mainly occurs through body fluids exchange, mostly by sexual contact, but blood, breastfeeding or vertical transmission have also been described. According to the WHO, 37.6 million people were infected by HIV in 2015 [74]. Fortunately, around 23% decrease in infections was registered from 2010 to 2019, but the treatment, diagnosis, and prevention remain as a global challenge, especially for developing countries with lack of resources. To date, antiretroviral therapy has shown an excellent outcome in the clinical management of AIDS. However, these drugs produce important side effects, including HIV resistance to the treatment [75]. 

**Figure 4:**
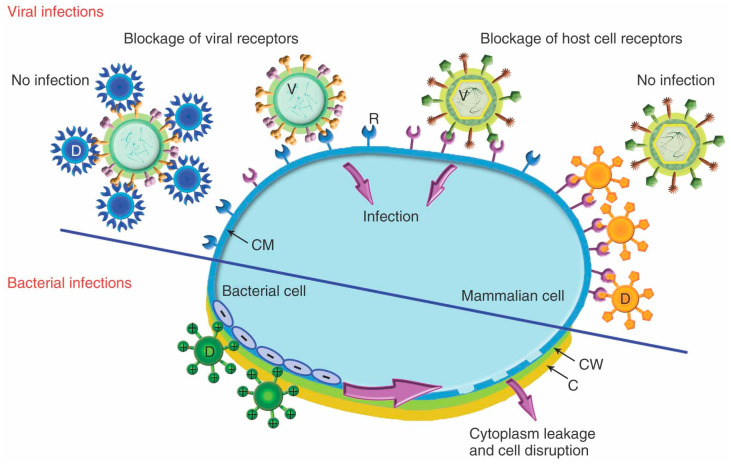
Proposed mechanism for the antiviral effect (**top**, through blockage of viral or host cell receptors) and antibacterial activity (**bottom**, through electrostatic interaction and subsequent disruption of bacteria membrane) of dendrimers. Reprinted with permission from Ref. [57], copyright © 2012 Wiley Periodicals, Inc.

Dendrimers represent an interesting alternative to minimize these side-effects and prevent the transmission of HIV and other viral or bacterial STIs, Figure 4 [76]. A promising approach relies on the use of dendrimers bearing anionic, sugar, or peptide moieties to prevent the entry of the virus in the target cell. These dendrimers block either the host cell or the viral receptors (Figure 4, top), such as the glycoproteins gp120 and gp41 located at the HIV envelope, two key proteins for the interaction and fusion of HIV with CD4 T cells. The most representative example is the anionic PLL dendrimer SPL7013 [77]. SPL7013 is a component of two approved and marketed products (Starpharma): VivaGel^®^ antiviral condom, for the treatment and prevention of HIV and HSV (herpes simplex virus); and VivaGel^®^ BV for bacterial vaginosis (a second product under Phase III clinical trial NCT01577537). Furthermore, it also shows significant activity against other viruses, such as the coronavirus SARS-CoV-2 [78], enabling a fast-track development of tools to fight COVID-19. Polyanionic carbosilane dendrimers are also promising microbicides against HIV infection, as shown in different animal models [79]. Besides their own antiviral activity, Muñoz-Fernández et al. showed that their combination with Tenofovir and Maraviroc (two antiviral agents) produce almost complete inhibition of HIV infection and transmission [80]. Carbosilane dendrimers are also efficient towards HIV-HSV coinfection [81] and can be employed in the development of fast diagnostic assays based on dendronized magnetic nanoparticles [82] tools. Dendrimers can also contribute to the design of efficient vaccines against HIV, such as the PEG-citrate G2 dendrimer bearing multiple HIV epitopes which produced a significant cellular immune response *in vivo* and a higher Th1 response compared to Th2 [83]. 

#### 3.1.2. Dendrimers and Dendritic Materials against Other Viral Infections

Besides HIV and STIs, dendrimers are effective towards other virus such as the Enterovirus A71 (EV71). EV71 belongs to the *Picornaviridae* family, associated with the hands–feet–mouth disease in children, a syndrome characterized by the presence of cutaneous vesicles and ulcerations and frequently with severe neurological manifestations [84]. Currently, neither vaccines nor therapies have been approved to prevent or treat EV71 infection, representing an important global problem but specially in the Asian southeast [85]. A tryptophan-decorated pentaerythritol dendrimer was especially active towards EV71 in some clinical isolates in the low nM–high pM range [86]. As EV71 is mainly transmitted through fecal–oral route, these dendrimers could be used as a prophylactic method after their oral administration, thus avoiding the transfer of EV71 from the gut to the bloodstream. Similar dendrimers have also shown a dual activity towards EV71 and HIV [87].

**Figure 5:**
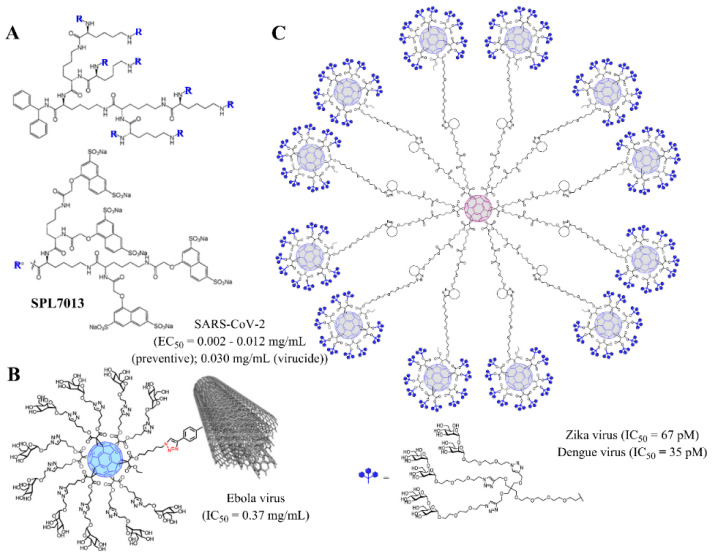
Examples of dendritic materials with potent antiviral activity and representative EC_50_/IC_50_ values. (**A**) SPL7013 dendrimer (VivaGel^®^), against SARS-CoV-2; (**B**) dendronized glycofullerene conjugated to multiwall carbon nanotubes, towards Ebola virus; (**C**) dendronized glycofullerene nanoballs, efficient towards Zika and Dengue viruses. Reprinted with permission from Refs. [84] (**B**) and [85] (**C**), Copyright 2018 and 2019, American Chemical Society.

Dendrimers and dendronized materials, such as fullerenes and carbon nanotubes, have also promising activity against other viruses like SARS-CoV-2 [78], Figure 5A; Ebola virus [88], Figure 5B; Zika and Dengue viruses [89], Figure 5C; HSV [90]; cytomegalovirus [91]; some flavivirus, such as the responsible of the Japanese encephalitis [92]; and different human or aviary flu viruses [93]. 

### 3.2. The Role of Dendrimers as Antibacterial Agents

Bacteria are unicellular prokaryote organisms with great implications in human health, as they compose the core of microbiota [94], but also in human disease. Certain bacterial populations can colonize and infect different tissues, leading to the development of a wide range of pathologies [95]. Antibiotics have long been one of the most effective solutions to fight against bacterial infections. However, their improper use drove a global public health problem: Bacterial resistance. Only in Europe, a total of 672,000 multiresistant bacterial infections were estimated, being responsible for up to 33,000 deaths in 2015 [96]. Dendrimers emerge as a potential solution, as they employ an unspecific mechanism that prevents the development of resistances (Figure 4 bottom). Some representative examples against resistant bacteria are herein collected.

#### 3.2.1. Dendrimers Against Biofilms: Example of *Pseudomonas Aeruginosa* Infections

Biofilms are one of the most important adaptive mechanisms of bacteria, enabling them to survive in an adverse environment. It is activated under different stress conditions, like limited oxygen or iron levels or the presence of some antimicrobial agents in sublethal concentrations [97]. *P. aeruginosa* represents one of the most important biofilm-forming bacteria, with outstanding impact in some chronic diseases like cancer [98] or cystic fibrosis [99]. This is the main problem associated with *P. aeruginosa* infection, especially in non-immunocompetent patients, hindering the clinical management. *P. aeruginosa* represents a clear example of how dendrimers can address resistant bacteria infection, including the inhibition of biofilm formation. 

**Figure 6:**
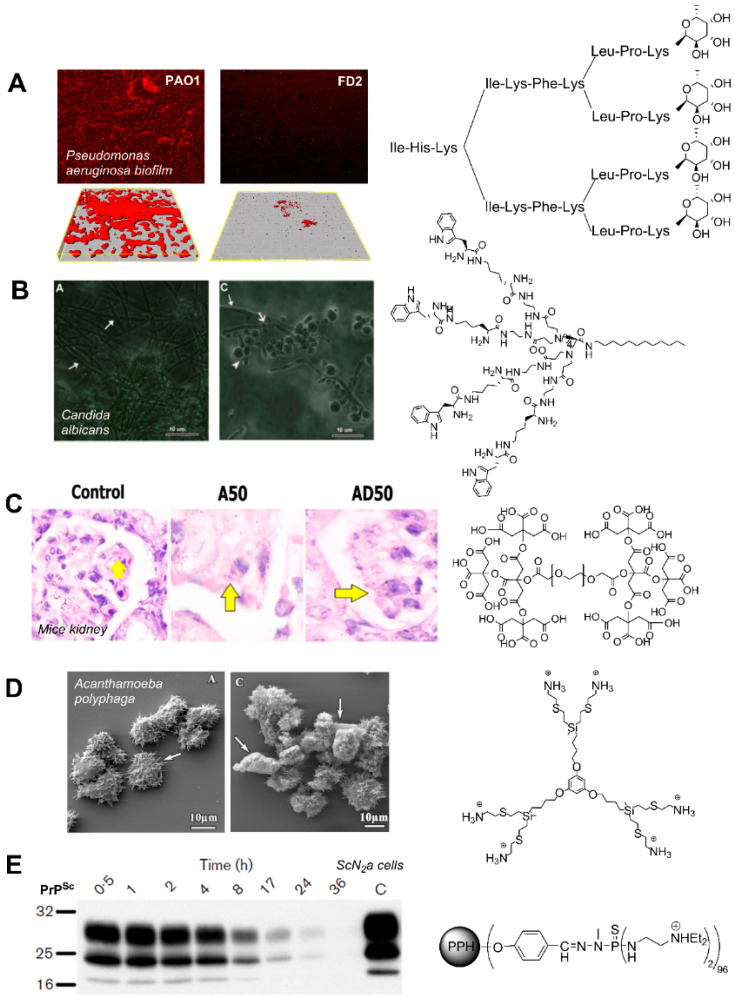
Examples of the antimicrobial activity of different dendritic molecules. (**A**) Inhibition of *Pseudomonas aeruginosa* biofilms by glycopeptide dendrimer FD2. Figure reprinted from ref. [100], Copyright 2008, with permission from Elsevier. (**B**) Effect on *Candida albicans* biofilm after treatment with a peptide dendrimer (16 µg/mL). Figure reprinted from Ref. [102], Copyright 2015, with permission from Elsevier. (**C**) Decrease in the number of *Leishmania Major* parasites in mice kidney after treatment with a dendritic polyester (A50) and amphotericin B loaded dendrimer (AD50). Figure reprinted by permission from ref. [103], Springer Nature, Copyright 2018. (**D**) Alterations of *Acanthamoeba polyphaga* trophozoites after treatment with a carbosilane dendrimer (2 mg/L). Figure reprinted from ref. [104]. (**E**) Evolution of PrP^Sc^ levels in ScN2a cells after treatment with a G4 PPH dendrimer (10 µg/mL). Reprinted with permission from Ref. [105], SGM, Copyright 2004.

It has been described that a bacterial specific lectine (LecB) plays a key role in biofilm formation by promoting the adhesion to cells [100]. Lectines are proteins which show a high specificity for sugars and their derivatives, being able to successfully recognize and agglutinate cells with glycosylated proteins or lipids. LecB binds to fucose, a mucin located in the epithelial mucosa, playing a key role in *P. aeruginosa* biofilm formation, in conjunction with LecA, specific to galactose, although this binding is weaker and less important [100]. In this context, some peptidic dendrimers have been developed to directly inhibit LecB-fucose interactions at low concentrations, such as FD2 (IC_50_ = 0.14 µM) depicted in Figure 6A. Dendrimers decorated with fucose-derived groups prevent the formation of *P. aeruginosa* biofilms (IC_50_~10 µM) and even disperse formed structures, by inhibiting the agglutination of the pathogen and acting as antimicrobial nanocarriers, thus increasing the efficacy of the established treatments [100,101].

#### 3.2.2. Additional Roles of Dendrimers in Bacterial Infection

One of the most representative examples of resistant bacteria is Gram-negative bacteria. These microorganisms present a peptidoglycan wall located between the inner and the outer membranes, which is responsible for the higher resistance of Gram-negative bacteria to immune system, and in case of lysis, promotes the release of proinflammatory substances known as lipopolysaccharides (LPS), exacerbating the infection [106]. This structure also confers Gram-negative bacteria resistance to some external agents through multiple mechanisms, which are heterogeneous between species [107]. Examples of common mechanisms of bacterial resistance are the expelling of toxic residues that eliminate antibacterial agents; a decrease in bacterial permeability, through the alteration of the membrane channels; or the production of antibiotic inactivating enzymes [108]. Cationic dendrimers may be a solution to fight against resistant bacteria, as they can bind efficiently to the negatively-charged walls, destabilize it by displacing sodium and calcium ions and increase the membrane permeability [109,110], Figure 4 bottom. However, despite their promising biocide activity, cationic dendrimers exhibit high toxicity towards mammal cells and require structural modifications to reduce their cytotoxicity, without affecting the efficacy [111]. Cationic antimicrobial peptides (AMP) arranged as Multiple Antigen Peptide (MAP) dendritic structures can also exert a potent antibacterial activity, decreasing the Minimum Inhibitory Concentration (MIC) and Minimum Bactericide Concentration (MBC) of the peptide alone, while dramatically increasing the peptide stability to proteolysis [112]. For example, a dendritic MAP structure based on the peptide QKKIRVRLSA effectively inhibited diverse Gram-negative bacteria (MIC = 4–8 g/mL for *E. coli* ATCC 25922, *P. aeruginosa* ATCC 27853, and a clinical isolate of *K. pneumoniae*) [112]. The higher local concentration of AMP allows a multivalent binding and enhances the destabilizing effect of the bacterial membrane. Dendrimers may also be used in the rapid diagnosis to discern between Gram-negative or Gram-positive bacteria, through a pH-dependent bacteria-selective aggregation occurring within 5 min of adding the dendrimer to a bacterial suspension [113]; and as carriers of different drugs such as vancomycin or agents like LED209, both specific to Gram-negative microorganisms [114,115].

Chorioamnionitis is an infection in the amniotic liquid, which may cause neurological problems in the fetus due to the production of proinflammatory cytokines, *Escherichia coli* being one of the most important etiological agents [116]. This disease often occurs due to the ascent of microorganisms from vagina to uterus, although other pathways have also been reported like transplacental infection, retrograde seeding from the peritoneal cavity through the fallopian tubes or accidental invasive procedures [117]. The use of antibiotics such as penicillins, cephalosporins, macrolides, and corticosteroids reduce the risk of developing chorioamnionitis [118]. In this sense, dendrimers can increase their efficacy. For example, a study conducted by Wang et al. (2010) in a Guinean pig model demonstrated that hydroxyl- and amino-functional PAMAM dendrimers successfully encapsulate ampicillin [119]. Both dendrimers significantly decreased the uterus cytokines, compared to the usual therapy, but the amino-dendrimer exhibited a higher toxicity. 

Dendrimers have been studied against other bacterial infections like *Chlamydia trachomatis*, increasing the efficacy of vaccines by conjugating a peptide mimic of a chlamydial glycolipid antigen to a G4-PAMAM-OH dendrimer [120]; some opportunistic agents, such as *S. aureus*, using different generation Gn-PAMAM-NH_2_ dendrimers [121]; or genital infections, through a sustained and localized delivery of amoxicillin in the cervicovaginal region by PAMAM-PEG dendritic hydrogels [122]. Overall, these studies show the potential role of dendrimers in bacterial infections, although a long road is still to cover, especially to decrease their cytotoxicity and increase their specificity.

### 3.3. The Role of Dendrimers as Antifungal Agents

Fungi are eukaryotic organisms responsible of a wide range of human infections. The prevalence of these diseases has increased in some countries, particularly in hospital areas and immunocompromised patients, *Candida*, *Cryptococcus*, *Pneumocystis*, and *Aspergillus* being the most representative families [123]. Data collected by the National Nosocomial Infections Surveillance System (NNIS) from January 1990 to April 1996 showed that up to 9% of nosocomial infections in EEUU were due to fungi, mainly *Candida* species [124]. However, current trends indicate the prevalence of *Aspergillus* in different European states [125].

The treatment of fungal infections is performed through antifungal (antimycotic) drugs, which produce some alterations in their cellular structures, thus inhibiting their development, viability and survival in a direct or indirect way. The most representative antifungal drugs include [126]: *Polyenes* (e.g., amphotericin B); *azoles* (e.g., imidazole, triazole); *allylamines*; *lipopeptides*; and *miscellaneous agents*, as griseofulvin, which inhibits microtubules and mitotic fuse, affecting cell division. Unfortunately, the resistance developed by some fungi may hinder the clinical management of these infections [127]. In addition, the great similarity between mammal and fungal cells can lead to cytotoxicity problems, being necessary to find molecules which selectively target fungal cells in a particular tissue [126]. In this context, the use of nanoparticles like dendrimers may be an effective method to carry all these substances, maintaining their benefits and reducing their side effects.

*Candida albicans*, which is responsible of more than half of total fungal infections around the world [128], is often treated with ketoconazole, a dual-action drug capable of inhibiting both ergosterol synthesis and the transformation of spores to micellar infectious forms [129]. However, ketoconazole is poorly water-soluble and can greatly benefit from the use of nanocarriers, which increase its bioavailability in the bloodstream. Gn-PAMAM-NH_2_ dendrimers improve the administration of ketoconazole (up to 16-fold increase of antifungal activity using G2 dendrimer, compared to the drug alone), being even more efficient when used as hydrogel formulation [129]; as well as clotrimazole (up to 32-fold increase with G2 dendrimer) due to its hydrophobic and electrostatic interactions [130]. Similarly, these dendrimers have significantly improved the antifungal activity of amphotericin B, overcoming the low water solubility and nephrotoxicity issues [131]. On the other hand, peptide dendrons have shown efficient anti-Candida activity per se [102]. The representative example shown in Figure 6B, which displays four tryptophan residues in the periphery and a dodecyl chain in the focal point, produced 100% growth inhibition at 16 µg/mL, as well as affected the biofilm viability and the hyphal and cell wall morphology.

On the other hand, dendrimer-assisted gene therapy can prevent fungal infections. Cationic PAMAM dendrimers, bearing –NH_2_, –NMe_2_, and –NMe_3_^+^ peripheral groups, were used with a ribozyme extracted from an intronic region of *C. albicans*, an RNA molecule capable to cut other RNA chain or even itself [132]. These dendrimers inhibited the catalytic activity of *Candida* ribozymes, with a generation-dependent activity (G4 > G3 > G2). However, the nature of the peripheral group did not produce a significantly different inhibition. Consequently, the construction of the dendrimer depended on the size of the RNA to inhibit and the charge ratio between dendrimer and RNA [133]. The use of RNA:dendrimer complexes may have multiple applications, such as inhibiting protein synthesis, splicing or even RNA delivery in an era where non coding RNA are beginning to be used, thus showing the potential of dendrimers in targeted therapy [134].

Unfortunately, the diagnosis of fungal diseases is currently a challenge. The current golden standard for the detection of these infections relies on poorly sensitive and invasive methods such as cell culture and histopathological study of the infected tissue [135]. New diagnostic tools are demanded, such as Polymerase Chain Reaction (PCR), immunoassays, or tests capable of detecting specific fungal antigens such as beta-glycans [136]. In this context, the use of nanostructures can play a key role in the development of new techniques that are more sensitive and effective in the early diagnosis of fungal infections. At present, very few studies report the use of dendrimers in the diagnosis of fungal diseases. Takano et al. (2003) performed cDNA microarray analysis using highly sensitive dendrimer-based technology in the detection of the rice pathogen *Magnaporthe grisea* and the stage of infection in this pathogen [137]. Another potential approach is the DendriChips^®^ technology (DENDRIS), relying on a phosphorous dendrimers coating which dramatically increases their sensitivity. This tool is capable of discerning up to 11 respiratory bacterial pathogens from a single sample [138], and could be refined and targeted to other types of infectious agents such as fungi. 

### 3.4. The Role of Dendrimers in Parasitic Diseases

Parasites are organisms characterized by the need of other living organism or “host” in order to survive. Parasites comprise an important variety of species of diverse complexity, from the simplest organisms such as protozoans to the more complex ones, such as plants [139]. Helminths and protozoans entail the main threat of human parasitosis; the clinical expression and its severity depend on the condition of the immune system of the host, as part of a tight interrelation [106,140]. Prevention could be the most efficient mechanism of controlling parasitic infections but, despite the considerable efforts, there are no effective vaccines against any of the main parasites. Accordingly, antiparasitic drugs are the pillar in protozoan control, when the simple prevention measures fail. However, the drug resistance of protozoans is becoming an alarming public health problem [141]. Dendrimers could be an effective tool in the early diagnosis or prevention of parasitosis, as well as a new treatment for some of these infections. 

#### 3.4.1. Dendritic Materials in Diagnosis and Prevention of Parasitic Infections

Protozoans comprise a diverse group of eukaryotic unicellular microorganisms that belong to *Protista* kingdom. Most common human infections caused by protozoans are related to *Plasmodium* spp. and *Toxoplasma gondii*, as well as *Trypanosoma* and *Leishmania* spp. Protozoan parasites are responsible for a considerable mortality and morbidity all over the world, that affect more than 500 million people [142]. Malaria is par excellence the main parasitic infection and it is caused by intracellular *Plasmodium* parasites transmitted by mosquitoes of genus *Anopheles*. Approximately, 40% of world population lives in areas where malaria is transmitted, causing 300–500 million infections and 2.7 million deaths per year. In specific regions, such as sub-Saharan Africa, children below 5 years-old conform 90% of the total deaths from malaria [143]. 

**Figure 7:**
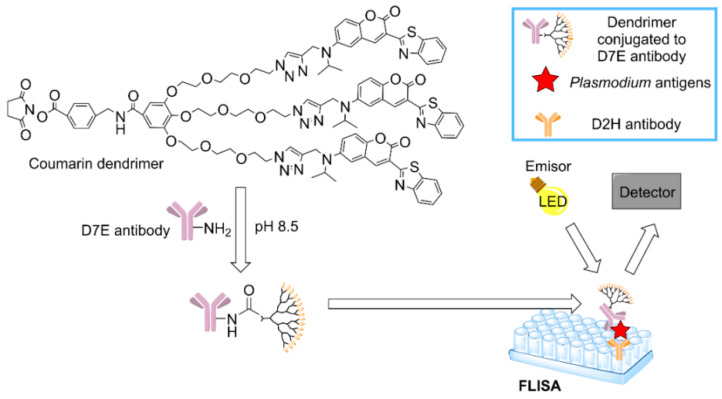
Mechanism to detect malaria antigens using the coumarin-derived dendrimer-based fluorescence-linked immunosorbent assay (FLISA) [145].

An important epidemiological study of the prevalence of malaria in Salomon Islands revealed the high rate of asymptomatic patients, highlighting the need for a diagnostic tool with high sensibility and specificity to detect *Plasmodium* [144]. This study relied on PCR and Rapid Diagnostic Tests (RDT), which are more sensible than a simple inspection with a microscope, but also far more expensive. In order to find a sensible detection method with a lower cost, a dendrimer-based assay was approved in South Korea [145]. The coumarin-derived dendrimer-based fluorescence-linked immunosorbent assay (FLISA) could detect two specific antigens of malaria: Histidine-rich protein II (HRP2) and lactate dehydrogenase (LDH). FLISA has good spectroscopic properties, such as photostability [146], and a better performance than traditional ELISA, enabling the quantification of the number of parasites in a sample even if they are present in low concentrations. Accordingly, FLISA method could be useful to detect asymptomatic cases at a modest price and with a high capacity. The process is depicted schematically in Figure 7. 

The role of dendrimers against helminthic *Schistosoma* parasites has also been tested. The disease, known as schistosomiasis [147], begins when the larvae form penetrates in the organism through the skin and settle in mesenteric and pelvic veins of the host, turning into the adult form. Here, the female parasites lay eggs, which could be eliminated through feces or urine or lead to complications like granulomas or intestinal, hepatosplenic and urogenital damage. This highlights the need for an early diagnosis. Wright et al. (2019) confirmed the promising activity of magnetic particles coated with G4-PAMAM-NH_2_ to concentrate the *Schistosoma* circulating anodic antigen (CAA), resulting in a 200-fold improvement in CAA limits of detection for lateral flow assays [148]. 

Preventive measures like vaccines are key to stop the impact of *Schistosoma* parasites in specific regions where they cause endemic infections. For example, infections by *S. haematobium*, *S. japonicum*, or *S. mansoni* affect over 200 million people worldwide [149]. Lysine-decorated PAMAM dendrimers showed excellent behavior as vaccine vector, enhancing the immunoreactivity and efficacy of DNA vaccine against *S. japonicum* infection [150]. Along the same lines, Anderson et al. (2016) developed a dendrimer-based vaccine platform which encapsulate antigen-expressing replicon mRNAs and generate a protective response towards others parasites such as *Toxoplasma gondii*, and relevant viruses like Ebola and H1N1 influenza, with a single dose [151]. The vaccine nanoparticle comprised an ionizable G1-PAMAM dendrimer, a lipid-anchored PEG and RNA. These studies show the role of dendrimers in the development of new generation vaccines against different infections.

#### 3.4.2. Dendrimers as Treatment of Parasitic Infections

Leishmaniasis is a parasitic disease produced by *Leishmania* protozoans that infects and multiplies in macrophage-rich organs and tissues of the reticuloendothelial system, mainly the liver and spleen. Estimations indicate an increase of 1.5 to 2 million cases per year [152]. For decades, leishmaniasis has been treated with Pentostam and Glucantime, leading to drug resistance and serious side effects like pancreatitis. Alternative broad-spectrum drugs with less toxicity, such as Amphotericin B, have been used but they present disadvantages such as the high cost, low solubility, the side effects, and its less efficacy as antiparasitic agent. Mannose-decorated G5-PPI dendrimers improved the activity of Amphotericin B for the treatment of Leishmaniasis, reducing the toxicity by increasing the targeting in macrophage-rich organs [153]. Other nanocarrier, a dendritic-linear-dendritic hybrid based on PEG and citric acid, Figure 6C, also improved the solubility of Amphotericin B (478 times) and reduced the *in vitro*/*in vivo* toxicity. It resulted as potent as free Amphotericin and Glucantime in reducing the parasite burden and number [103].

Toxoplasmosis is a zoonosis caused by the ingestion of the parasite *Toxoplasma gondii*. This disease has a chronic and silent course in the majority of population without immune system disorders, mainly causing symptoms such as low fever and muscular pain. This infection is usually treated with sulfadiazine [154], which has two disadvantages: it requires a high dose of the drug, which can produce severe side effects like high fever or allergic reactions; and it cannot reach target tissues where the parasite is typically localized. Cationic G4 PAMAM dendrimers and anionic G4.5 dendrimers efficiently solubilize sulfadiazine (up to 30 molecules per dendrimer) and improve the penetration into the parasite, thus greatly reducing the required sulfadiazine dose and localizing the drug in muscle and brain, where *T. gondii* is usually present [155]. The main dendrimer–drug interactions found were electrostatic, for cationic dendrimers, and hydrogen bonding, for anionic counterparts. Furthermore, the anionic dendrimer showed intrinsic antiparasitic effect. 

Other successful examples of antiparasitic dendrimers include PEGylated PLL dendrimers coated with chondroitin sulfate A, as targeted unimolecular micelles for the delivery of the antimalarial drug chloroquine phosphate [156]; and PAMAM dendrimers decorated with ethynil estradiol against *Trypanosoma cruzi*, the parasite responsible for Chagas disease, where the G2 dendrimer is 8 times more effective than benznidazole at 24 h and 48 h (IC_50_ = 1.25 µM) [157].

### 3.5. Dendrimers against Amoeba Infections

Amoebae are eukaryotic protozoa extensively distributed in nature and human habitats, often acting as a host and reservoir of other microorganisms like giant viruses and some class of bacteria [158,159]. Amoebae infecting humans are classified as parasitic, such as *Entamoeba* organisms, or opportunistic free-living amoebae, such as *Acanthamoeba* spp., *Balamuthia mandrillaris*, *Sappinia diploidea*, and *Naegleria fowleri* (known as the brain-eating amoebae) [160]. Free-living amoebae do not require human infection for their life cycle, which presents two stages: trophozoite (active form) and cyst (inactive form). *Acanthamoeba* and other free-living amoebae are responsible of serious diseases such as keratitis, encephalitis, and infections in immunocompromised patients in the central nervous system, skin, and lungs. Fortunately, amoebae infections are relatively rare, although the high mortality associated with meningoencephalitis are of concern, mainly due to the late diagnosis and the lack of effective antimicrobial treatments [161]. 

Low generation cationic carbosilane dendrimers bearing ammonium or biguanide moieties have shown strong amoebicidal activity against trophozoites and cysts of *Acanthamoeba* spp., Figure 6D (IC_50_ = 2.4 mg/L; minimum cysticidal concentration MCC = 256 mg/L) [104,162]. Furthermore, they exhibit a synergistic effect with traditional drugs such as chlorhexidine, decreasing the required drug concentration 5–10 times using G1 dendrimers [163]. The dendrimers target the amoeba membrane and produce inhibition and cell death. In this context, dendrimers could be a promising therapeutic alternative to properly manage amoebae infections. 

### 3.6. Dendritic Materials against Prionic Diseases

Prions (PrP) are infectious pathogens that cause neurodegenerative transmissible diseases such as spongiform encephalopathies, after a long period of incubation from 2 to 10 years [164,165]. PrP^Sc^ are glycoproteins with an abnormal folding that are originated from a conformational change of normal prion proteins (PrP^C^), acquiring pathological properties [166]. Prion diseases can occur for two reasons: the infectious prion agent can transmit the pathological folding to the PrP^C^; or the PRNP gen mutates, leading to a genetic variation of the prion disease [167]. Infectious prion diseases have gained importance since the epidemic bovine spongiform encephalopathy in United Kingdom in 1986 that was transmitted to humans as a variation of the Creutzfeldt-Jakob prion disease [168,169]. The spongiform encephalopathies are named after the shape the brain acquires at final stages of the disease, caused by an increase of vacuoles and the holes that appear in the tissue. It is a rare but severe pathology, which leads to neuronal loss and, eventually, dementia.

Nowadays, there are some pharmacological interventions that delay the progression of prion diseases; however, there is no effective treatment to stop or avoid it in a significant way [170]. In this field, nanomaterials such as dendrimers can exert a relevant activity, preventing the conversion of PrP^C^ to PrP^Sc^ [171]. Due to the high affinity of amine groups to prions, phosphorus dendrimers decorated with tertiary amines in their surface are promising agents in the therapy against prion diseases, Figure 6E [105]. These dendrimers showed an inhibitory effect in the generation of prions (IC_50_ = 10 (G3), 1.5 (G4) and 5 (G5) μg/mL) and they had anti-infectious action *in vitro* and *in vivo* for some spongiform encephalopathies, some of them causing Creutzfeldt-Jakob disease [105]. Other dendritic families with even higher anti-prion activity include G4-PAMAM-NH_2_ and G4-PPI-NH_2_ (both IC_50_ = 80 ng/mL) [172,173]. For example, Ottaviani et al. (2012) showed that PPI glycodendrimers, comprising maltose or maltotriose units, prevented the aggregation of PrP and Aβ(1-28) proteins due to the interference of dendrimers with the latency phase (nucleation) of the prion protein [174]. 

Dendrimers can also be useful for the diagnosis of prion infections. Usually, ELISA is used to determine whether the causing agent of the disease is a prion, but this method has important limitations as it cannot distinguish between prion chains [175]. The nature of the prion agent will determine the etiology and course of the disease, changing the pathology parameters like the incubation period or the type of lesion [176]. Importantly, different generation PAMAM and PPI dendrimers were capable of distinguishing between the different types of prions [177]. This study demonstrated that the susceptibility of a prion towards a particular dendrimer could be used to diagnose and predict the course of a disease. On the other hand, Korri-Youssoufi et al. developed an electrochemical biosensor comprising multiwalled carbon nanotubes modified with dendrimers and aptamers to detect prion proteins with a high sensibility (min. 0.5 pM) [178]. In conclusion, dendrimers constitute a promising research line for fighting against prion diseases as they could slow down their progression and allow a reliable diagnosis of the responsible etiological agent.

### 3.7. Other Applications of Dendrimers which May Benefit the Management of Infectious Diseases

Dendritic nanosystems could have a principal role as excipients in the pharmaceutical industry, providing that they are compatible, safe and effective nanocarriers in several administration routes [179]. For example, ocular administration is still a challenge due to the unique physiology of the eye and the presence of numerous barriers. In this context, recent studies showed that dendrimers improved the time that a drug remains in the cornea after topical administration and they supply directed and sustained neuroprotection for retina [180]. Moreover, dendrimers could act as DNA vectors, providing safety and reducing cytotoxicity compared to the viral vectors and intraocular injections used at present [53].

Dendritic polymers can easily improve the properties of different materials, such as cotton, to generate antimicrobial activity. For example, the addition of amine-functional dendrimers confers antibacterial activity against Gram-positive bacteria (*E. coli*, *P. aeruginosa*) and Gram-negative bacteria (*S. aureus*) and fungi (*C. albicans*) [181,182], even after several washing cycles.

Sepsis is a life-threatening response to an infection, which happens when the immune system overreacts and starts to damage the patient’s own tissues and organs. Peptide-decorated PAMAM dendrimers inhibited the acetylation of transforming growth factor β-induced protein, thus improving the mortality and organ damage in the septic mouse model [183]. Additionally, gadolinium-containing G4 dendrimers can be used as MRI diagnostic and prognostic biomarker of sepsis-induced acute renal failure [184]. These studies open up a field in the treatment and diagnosis of sepsis with a not very high cost and that could be protective against infectious pathogens in certain circumstances like during hospital stays, surgeries or risky exposures.

The design of metallodendrimers is a promising field to explore, which can open new avenues in the fight against infectious diseases [185]. Dendrimers are excellent platforms to control the attachment of a great variety of metal complexes with antimicrobial activity, such as silver(I), copper(II), and zinc(II), among others. The resultant metallodendrimers often exhibit a synergistic activity and improve the therapeutic response of the dendrimer or the metal complex alone. For example, Muñoz-Fernández et al. (2015) demonstrated the impact of a single metal ion in the prevention of HIV infection [186]. A carboxylate-decorated G2 PPI dendrimer, which inhibited HIV-1 infection of Hec-1A cells around 30%, reached 90% inhibition by attaching a single Cu(II) ion through its ethylenediamine core. The remarkable control on the dendritic structure enables an optimized antimicrobial activity, difficult to accomplish with other nanomaterials. The reader is referred to a recent review on this topic [185].

## 4. Conclusions

Dendrimers are an innovative tool in the treatment, prevention and diagnosis of serious infectious pathologies, as summarized in Table 1. They emerge as a unique opportunity to overcome problems of traditional approaches, such as microbial resistance. Nevertheless, the transition from the laboratory to the clinical practice still requires facing several challenges, such as the big scale production, the batch-to-batch consistency, the purity for clinical tests, or the regulatory obstacles. It should be kept in mind that most investigations have been conducted in experimental models *in vitro* or in animal models, so it is not possible to extrapolate these results to humans yet. However, the outstanding results obtained by VivaGel^®^ in the prevention of viral and bacterial infections predict a brilliant future of dendritic materials in the fight against infectious diseases.

## Figures and Tables

**Table 1:** Overview of the potential of dendrimers and dendritic materials in the fight against infectious diseases, including selected examples.

Disease	Strategy	Examples and Advantages	Ref.
Viral	Treatment	Dual microbicide and drug nanocarrier against HIV	[80]
		Microbicide against flavivirus (gene carrier)	[92]
	Diagnosis	Rapid diagnosis of HIV-1	[82]
	Prevention	Inhibition of entry into target cell: HIV, HSV, HCMV, IAV, SARS-CoV-2, Ebola, Zika and Dengue	[77,78,79,81,88,89,91,93]
		Nanocarrier in HIV-1 vaccine	[83]
		Inhibition of EV71 transfer from gut to bloodstream	[88]
Bacterial	Treatment	Inhibitions of *P. aeruginosa* biofilm	[100,101]
		Intrinsic bactericide effect	[109,111,112]
		Drug nanocarrier against Gram-positive/negative bacteria	[114,115,119]
	Diagnosis	Rapid diagnosis. Discern Gram-positive/negative bacteria	[113]
	Prevention	Nanocarriers in peptide vaccines	[120]
Fungal	Treatment	Drug nanocarrier for improved activity against *C. albicans*	[129,130,131]
	Diagnosis	High-sensitive detection of fungi genes	[137]
	Prevention	Inhibition of catalytic activity of *Candida* ribozymes (RNA vector)	[132]
Parasitic	Treatment	Drug nanocarrier for improved treatment of Leishmania, toxoplasmosis or malaria	[153,155,156]
		Antiparasitic activity per se for Chagas disease	[157]
	Diagnosis	High-sensitive detection of *Plasmodium* parasite, *Schistosoma* ACC	[145,148]
	Prevention	DNA/RNA vector for high immunoreactivity vaccines	[150,151]
Amoeba	Treatment	Antimicrobial activity to both trophozoite and cyst forms of *Acanthamoeba* spp.	[104,162,163]
Prion	Treatment/Prevention	Prevention of the conversion of PrP^C^ to PrP^Sc^	[171]
		Prevention of PrP aggregation	[174]
	Diagnosis	Detect and distinguish between prion diseases	[177]
